# Supplementary material for: Changes in HMO Concentrations throughout Lactation: Influencing Factors, Health Effects and Opportunities
Source: Nutrients. 2021 Jun 30;13(7):2272. doi: 10.3390/nu13072272 (PMC8308359; doi:10.3390/nu13072272)
Supplement: Supplementary file 1 [file nutrients-13-02272-s001.zip › nutrients-1253085-supplementary.pdf]

Supplementary Materials

The selection of manuscripts to be included in this review was based on the criteria published in Thurl et al, 2017 systematic review (Table S1).

Table S 1 Parameters for the selection of manuscripts included in this review.

Parameter	Inclusion criteria	Exclusion criteria
Population	Milk samples from individual healthy mothers	Animal studies
	Documented duration of pregnancy	Pooled milk samples
	Documented lactation days	
Intervention	None	
Outcomes	Absolute concentrations of a single HMO	Relative concentrations
	Mean values	Median values
	Single HMO concentration values with n ≥ 2 at given lactation period (ie, values from at least 2 mothers were required)	Concentrations of mixtures of HMOS
Study design	Original articles from peer-reviewed journals	Abstracts, monographs

Table S 2 Concentrations of individual HMOs in human colostrum, transitional, and mature milks from mothers with either secretor positive, or unknown secretory status. The concentration (mg/L) of the HMOs was shown as mean ± standard deviation or mean (range).

**(a) Colostrum**	**Day 1**	**Day 2**			**Day 3**			**Day 0-4**	**Day 0-5**	**Day 1-7**	
**Reference**	[44,45]	[44,45]	[25]	[21]	[44,45]	[43]	[26]	[2]	[34]	[31]	[24]
**Year**	2007 & 2008	2007 & 2008	2018	2019	2007 & 2008	2010	2018	2017	2015	2017	2019
**Company**			Fonterra	Nestle			Danone				Sanyuan
**Country**	Japan	Japan	Malaysia /Chinese	7 European countries	Japan	Germany	USA	Various	USA	Spain	China
**2'-FL**	2490 ± 1220	2010 ± 1070	2249 ± 1764	3691 ± 1941	1580 ± 730	4130	3750 ± 100	3230 ± 610	2652 ± 2222	2210 (0-4690)	1705 1101
**3-FL**	260 ± 140	280 ± 260	429 ± 419	422 ± 453	200 ± 130	240		240 ± 100	444 ± 513	750 (0-1190)	353 ± 305
**LNDFH I**	1270 ± 540	1870 ± 1550		1232 ± 519	1410 ± 660	1120	2100 ± 60	860 ± 240			798 ± 570
**LNDFH II**	17 ± 18	20 ± 25			19 ± 28	100		80 ± 90		60 (10 -250)	
**LDFT**	420 ± 420	280 ± 300		607 ± 558	190 ± 140	490	360 ± 10	520 ± 270	159 ± 152	180 (0-400)	
**LNT**	890 ± 430	1440 ± 700	2393 ± 2192	912 ± 802	1450 ± 730		480 ± 0	660 ± 410	1054 ± 984	840 (620-1600)	1123 ± 77
**LNnT**	400 ± 90	540 ± 140	1420 ± 1032	307 ± 132	420 ± 150			770 ± 830	255± 113	310 (140-450)	616 ± 239
**LNFP I**	1470 ± 1010	2080 ± 1670	3563 ± 1920	1928 ± 903	1670 ± 1030	2000	1810 ± 30	1570 ± 300	1409 ± 1153	950 (0-1300)	1509 1032
**LNFP II**	380 ± 240	450 ± 260		422 ± 518	420 ± 330	140		220 ± 190	401 ± 461	150 (0-1510)	365 ± 409
**LNFP III**				445 ± 166		340		260 ± 290	359 ± 188	380 (260-560)	
**LNFP V**				108 ±103				3 ± 1223			60 ± 75
**3′-SL**	362± 103	269 ± 70	222 ± 83	254 ± 90	259 ± 80	350	110 ± 10		228 ± 63	230 (160 - 330)	228 ± 78
**6′-SL**	342 ± 120	371 ± 115	651 ± 411	543 ± 168	397 ± 86	1310	340 ± 30	380 ± 90	520 ± 152	680 (500-800)	1175 ± 49
**LST a**	107 ± 85	155 ± 118	160 ± 111		162 ± 111	60		120 ± 400		150 (110-240)	
**LST b**	68 ± 22	64 ± 25		79 ± 40	61 ± 27	50				40 (20 - 50)	
**LST c**	659 ± 297	707 ± 261	1326 ± 641	497 ± 218	693 ± 243	480		480 ± 150		380 (290-440)	743 ± 255
**DSLNT**	480 ± 126	447± 110		405 ± 178	459 ± 151	290				380 (240-540)	804 ± 721

**(a) Colostrum**	**Day 1**	**Day 2**			**Day 3**			**Day 0-4**	**Day 0-5**	**Day 1-7**	
**Reference Year**	[44,45] 2007 & 2008	[44,45] 2007 & 2008	[25] 2018	[21] 2019	[44,45] 2007 & 2008	[43] 2010	[26] 2018	[2] 2017	[34] 2015	[31] 2017	[24] 2019
**Company**			Fonterra	Nestle			Danone				Sanyuan
**Country**	Japan	Japan	Malaysia /Chinese	7 European countries	Japan	Germany	USA	Various	USA	Spain	China
**3′-GOS/3'-GL**									419 ± 131		17± 11
**6′-GOS /6'-GL**				132 ± 47							73 ± 30
**DFLac**											
**A-tetra**											
**FLNH/FLNH I/FLNH II**								590 ± 450			
**TFLNH**								2730 ± 1350			
**DFLNT**											
**LNnFP**											
**LNnFP-V**				37 ± 16							
**LNH**							80 ± 10	70 ± 100			
**LNnH**								180			
**LNnDFH**				113 ± 73							
**MFLNH I/2-FLNH**						210	110 ± 10				
**MFLNH III/3-FLNH**				201 ± 155		70					
**2'3'-DF-LNH**						350					
**DFLNH/DFLNHa/DFLNH I/DFLNH II**				162 ± 96				2800 ± 5480			
**DFLNnH**								510			
**DSL**			5.3 ± 3.6								
**SLNFP I**	76 ± 55	80 ± 37			78 ± 65						

**3'S3FL**	148 ± 44	156 ± 64	23.4 ± 32.2		165 ± 44
**SLNFP II**					
**6'SLN**			21.6 ± 22.2		
**DSLNH**					
**FDSLNH**					

**(b) Transitional**	**Day 5-15**									
**Reference Year**	[43] 2010	[43] 2010	[42] 2010	[34] 2015	[32] 2016	[2] 2017	[31] 2017	[25] 2018	[23] 2019	[24] 2019
**Company**					Nestle			Fonterra	Fonterra	Sanyuan
**Country**	Germany (8 d)	Germany (15 d)	Samoa (5-10 d)	USA (14 d)	China (5-11 d)	Various (5-10 d)	Spain (8-15 d)	China (14 d)	UAE (5-15 d)	China (8-15 d)
**2’-FL**	3370	3040	220 ± 370	2061 ± 1416	2000 ± 1400	3050 ± 710	2340 (0-3860)	1281 ± 1050	2021 ± 1776	1507 ± 898
**3-FL**	260	380	1670 ± 820	933 ± 567	490 ± 600	270 ± 120	950 (0-1430)	543 ± 501	581 ± 868	476 ± 397
**LNDFH I**	1300	1460	750 ± 680			690 ± 290				777 ± 548
**LNDFH II**	170	230	860 ± 440			160 ± 120	120 (40-200)			
**LDFT**	330	480	70± 60	178 ± 184		450 ± 330	220 (0-340)			
**LNT**			3900 ± 1860	870 ± 623	880 ± 530	920 ± 650	1000 (770-2570)	1979 ± 738	1429 ± 693	1207 ± 599
**LNnT**			460 ± 360	149 ± 71	180 ± 85	1080 ± 1220	200 (0-1260)	1033 ± 445	765 ± 350	329 ± 153
**LNFP I**	2250	1640	280 ± 580	862 ± 734	910 ± 740	1910 ± 440	870 (0-1550)			1147 ± 802
**LNFP II**	230	290		359 ± 384		360 ± 190	200 (0-1260)			399 ± 377
**LNFP III**	340	370		248 ± 111		340 ± 410	330 (270-420)			
**LNFP V**					41 ± 49					60 ± 69
**3′-SL**	300	270	163 ± 105	165 ± 38	110 ± 35		200 (140 - 300)	100 ± 42	226 ± 107	154 ± 36
**6′-SL**	1770	1570	343 ± 235	558 ± 140	330 ± 140	470 ± 110	640 (530-970)	592 ± 219	621 ± 212	1297 ± 426

**LST a**	90	50	78 ± 60	90 ± 30	160 (120-230)	127 ± 86	104 ± 46	
**LST b**	60	70	84 ± 43		40 (20-150)			
**LST c**	60	310	620 ± 458	500 ± 100	370 (230-510)	941 ± 528	488 ± 224	367 ± 147
**DSLNT**	60	440	638 ± 484		320 (230-490)			644 ± 552

**(b) Transitional**	**Day 5-15**									
**Reference Year**	[43] 2010	[43] 2010	[42] 2010	[34] 2015	[32] 2016	[2] 2017	[31] 2017	[25] 2018	[23] 2019	[24] 2019
**Company**					Nestle			Fonterra	Fonterra	Sanyuan
**Country**	Germany (8 d)	Germany (15 d)	Samoa (5-10 d)	USA (14 d)	China (5-11 d)	Various (5-10 d)	Spain (8-15 d)	China (14 d)	UAE (5-15 d)	China (8-15 d)
**3′-GOS/3'-GL**				338 ± 151						13.2 ± 13.1
**6′-GOS /6'-GL**										34.3 ± 19.2
**DFLac**										
**A-tetra**					13 ± 9.5					
**FLNH/FLNH I/FLNH II**						730 ± 510				
**TFLNH**						3050 ± 1350				
**DFLNT**										
**LNnFP**					11 ± 9					
**LNnFP-V**										
**LNH**						120 ± 100				
**LNnH**			160 ± 110			100				
**LNnDFH**										
**MFLNH I/2’-FLNH**	350	230								
**MFLNH III/3-FLNH**	180	220								
**2'3-DF-LNH**	410	400								
**DFLNH/DFLNHa/DFLNH I/DFLNH II**						2810 ± 5480				
**DFLNnH**						320				
**DSL**								3.1 ± 5.3	2.2 ± 2.3	
**SLNFP I**										
**3'S3FL**								5.7 ± 5.9	19 ± 21	
**SLNFP II**										
**6'SLN**								5.7 ± 4.1	15 ± 15	

**DSLNH**	
**FDSLNH**	

**(c1) Mature**	**1 month**									
**Reference Year**	[43] 2010	[65] 2010	[34] 2015	[32] 2016	[2] 2017	[31] 2017	[30] 2017	[25] 2018	[24] 2019	[21] 2019	[21] 2019
**Company**				Nestle			Nestle	Fonterra	Sanyuan	Nestle	Nestle
**Country**	Germany (22 d)	Germany (1mth)	USA (28 d)	China (12-30 d)	Various (11-30 d)	Spain (16-30 d)	Singapore	China	China (28-34 d)	7 European countries (17 d)	7 European countries
**2'-FL**	3020	2960	1753 ± 1382	1900 ± 1200	2830 ± 500	2190 (0-3860)	2170 ± 832	1371 ± 1121	1399 ± 860	2627 ± 1028	2450 ± 935
**3-FL**	440	420	767 ± 654	570 ± 480	340 ± 90	1050 (0-1170)		894 ± 718	732 ± 545	594 ± 554	720 ± 608
**LNDFH I**	1550	1360			690 ± 200				626 ± 441	1275 ± 548	1105 ± 452
**LNDFH II**	260	240			140 ± 80	110 (60-250)					
**LDFT**	360	370	140 ± 165		370 ± 230	190 (0-350)				349 ± 379	277 ± 231
**LNT**			750 ± 481	620 ± 340	760 ± 410	1010 (770-2100)	979 ± 394	1225 ± 553	651 ± 316	1213 ± 720	1009 ± 591
**LNnT**			113 ± 71	120 ± 67	630 ± 850	180 (110-230)	263 ± 99	708 ± 299	237 ± 143	177 ± 97	153 ± 80
**LNFP I**	1720	1480	546 ± 512	540 ± 400	1370 ± 290	920 (0-1560)		1181 ± 578	701 ± 650	1431 ± 798	1071 ± 627
**LNFP II**	300	240	367 ± 350		320 ± 110	190 (10 - 1240)			275 ± 250	595 ± 630	549 ± 532
**LNFP III**	370	370	222 ± 77		320 ± 240	310 (190-460)				320 ± 141	311 ± 98
**LNFP V**				39 ± 41	80 ± 860				39 ± 41	124 ± 117	112 ± 99
**3′-SL**	260	270	146 ± 32	94 ± 25		180 (140-220)	217 ± 74	108.1 ± 37.4	651 ± 316	149 ± 38	141 ± 35

**6′-SL**	1420	1350	368 ± 108	250 ± 93	380 ± 90	650 (470-780)	561 ± 200	365 ± 160	736 ± 450	649 ± 189	465 ± 162
**LST a**	30	30			70 ± 50	180 (110-230)		58 ± 40			
**LST b**	90	100				50 (20-230)				80 ± 40	77 ± 38
**LST c**	250	240			240 ± 110	290 (190-440)		159 ± 111	173 ± 132	258 ± 128	148 ± 72
**DSLNT**	410	410				310 (220-510)			336 ± 222	385 ± 164	290 ± 135

(c1) Mature	1 month										
**Reference Year**	[43] 2010	[65] 2010	[34] 2015	[32] 2016	[2] 2017	[31] 2017	[30] 2017	[25] 2018	[24] 2019	[21] 2019	[21] 2019
**Company**				Nestle			Nestle	Fonterra	Sanyuan	Nestle	Nestle
**Country**	Germany (22 d)	Germany (1 month)	USA (28 d)	China (12-30 d)	Various (11-30 d)	Spain (16-30 d)	Singapore	China	China (28-34 d)	7 European countries (17 d)	7 European countries
**3′-GOS/3'-GL**			257 ± 76						9.87 ± 8.40		
**6′-GOS /6'-GL**									22.3 ± 13.6	132 ± 47	26 ± 10
**DFLac**											
**A-tetra**				27 ± 42							
**FLNH/FLNH I/FLNH II**					460 ± 310						
**TFLNH**					3030 ± 960						
**DFLNT**											
**LNnFP**				8.1 ± 4.7							
**LNnFP V**										28 ± 10	28 ± 9
**LNH**					100 ± 90						
**LNnH**					90						
**LNnDFH**											58 ± 53
**MFLNH I/2-FLNH**		240									
**MFLNH III/3-FLNH**		200								416 ± 208	358 ± 192
**2'3-DF-LNH**	330	400									
**DFLNH/DFLNHa/DFLNH I/DFLNH II**					2700 ± 3880					278 ± 163	227 ± 147
**DFLNnH**					120						
**DSL**								1.0 ± 1.2			
**SLNFP I**											
**3'S3FL**								8.6 ± 10.2			
**SLNFP II**											
**6'SLN**								5.5 ± 3.5			

**DSLNH**	
**FDSLNH**	

**(C2) Mature milk**	**2 months**								**3 months**			
**Reference Year**	[43] 2010	[32] 2016	[2] 2017	[30] 2017	[25] 2018	[25] 2018	[21] 2019	[26] 2018	[43] 2010	[2] 2017	[25] 2018	[21]2019
**Company**		Nestle		Nestle	Fonterra	Fonterra	Nestle	Danone			Fonterra	Nestle
**Country**	Germany	China (1-2 months)	Various (1-2 months)	Singapore	China	Malaysia	7 European countries	USA (42 d)	Germany	Various (2-3 months)	China	7 European countries
**2'-FL**	2820	1700 ± 1100	2390 ± 710	1764 ± 635	1176 ± 1019	1286 ± 1034	2075 ± 840	2480 ± 130	2590	2210 ± 710	984 ± 894	1819 ± 739
**3-FL**	560	720 ± 550	640 ± 150		1158 ± 864	762 ± 597	970 ± 692		670	670 ± 120	1366 ± 942	1140 ± 777
**LNDFH I**	1020		1100 ± 290				842 ± 327	1930 ± 50	1050	990 ± 290		719 ± 285
**LNDFH II**	190		190 ± 130						170	180 ± 130		
**LDFT**	380		380 ± 460				280 ± 155	240 ± 10	480	350 ± 230		273 ± 131
**LNT**		370 ± 220	1010 ± 530	633 ± 324	851 ± 319	1217 ± 651	700 ± 416	510 ± 30		730 ± 530	947 ± 602	599 ± 400
**LNnT**		83 ± 43	560 ± 1220	166 ± 72	569 ± 226	609 ± 285	128 ± 80			760 ± 1230	513 ± 419	108 ± 67
**LNFP I**	1060	340 ± 240	830 ± 450		950 ± 397	1660 ± 494	611 ± 423	580 ± 30	940	830 ± 440	1177 ± 679	469 ± 373
**LNFP II**	180		240 ± 190				474 ± 402		170	210 ± 150		433 ± 332

**LNFP III**	400		400 ± 410				358 ± 110		440	440 ± 410		353 ± 92
**LNFP V**		26 ± 25					91 ± 72					85 ± 66
**3′-SL**	230	80 ± 22		195 ± 60	99 ± 21	112 ± 28	129 ± 31	120 ± 0	240		114 ± 29	130 ± 35
**6′-SL**	630	140 ± 81	300± 150	280 ± 116	222 ± 105	251 ± 132	231 ± 101	250 ± 20	490	140 ± 130	137 ± 55	151 ± 87
**LST a**	10		10± 60		43 ± 32	60 ± 31			10	20 ± 50	40 ± 28	
**LST b**	80						64 ± 33		80			57 ± 31
**LST c**	110		130 ± 90		152 ± 118	130 ± 77	70 ± 47		90	110 ± 110	85 ± 67	44 ± 42
**DSLNT**	230						169 ± 83		210			136 ± 72

(C2) Mature milk	2 months								3 months			
Reference Year	[43] 2010	[32] 2016	[2] 2017	[30] 2017	[25] 2018	[25] 2018	[21] 2019	[26] 2018	[43] 2010	[2] 2017	[25] 2018	[21]2019
Company		Nestle		Nestle	Fonterra	Fonterra	Nestle	Danone			Fonterra	Nestle
Country	Germany	China (1-2 months)	Various (1-2 months)	Singapore	China	Malaysia	7 European countries	USA (42 d)	Germany	Various (2-3 months)	China	7 European countries
3′-GOS/3'-GL												
6′-GOS /6'-GL							18 ± 9					14 ± 7
DFLac												
A-tetra		25 ± 36										
FLNH/FLNH I/FLNH II												
TFLNH												
DFLNT												
LNnFP		7.9 ± 4.1										
LNnFP V							31 ± 12					28 ± 8
LNH			80 ± 80					160 ± 10		120 ± 100		
LNnH			130							280		
LNnDFH							55 ± 31					57 ± 29
MFLNH I/2’-FLNH	130							110 ± 10	100			
MFLNH III/3-FLNH	120						208 ± 127		90			143 ± 94
2'3-DF-LNH	230								180			
DFLNH/DFLNHa/DFLNH I/DFLNH II			2840 ± 5480				120 ± 97					98 ± 80
DFLNnH												

**DSL**	0.7 ± 1.6						1.6 ± 3.9	
**SLNFP I**								
**3'S3FL**	8.5 ± 9.1	5.5 ± 3.5					7.5 ± 4.8	
**SLNFP II**								
**6'SLN**	5.5 ± 2.1	5.9 ± 1.3					3.8 ± 2.6	
**DSLNH**								
**FDSLNH**								

**(C3) Mature milk**	**4 months**					**6 months**		
**Reference Year**	[42] 2010	[32] 2016	[30] 2017	[27] 2018	[25] 2018	[25] 2018	[25] 2018	[23] 2019
**Company**		Nestle	Nestle		Fonterra	Fonterra	Fonterra	Fonterra
**Country**	Samoa (22-155d)	China (2-4 mth)	Singapore	Canada (Caucasian and Asian mothers) (3-4 mths)	China	China	Malaysia	UAE
**2’-FL**	690 ± 810	1300 ± 900	1376 ± 594	2256 ± 1846	866 ± 891	704 ± 752	1003 ± 803	997 ± 885
**3-FL**	2350 ± 1390	1100 ± 610		267 ± 171	1427 ± 892	1476 ± 790	1146 ± 869	1194 ± 106
**LNDFH I**	1220 ± 1710							
**LNDFH II**	700 ± 780							
**LDFT**	140 ± 190							
**LNT**	1310 ± 590	290 ± 170	407 ± 200	1047 ± 479	866 ± 443	785 ± 497	867 ± 426	504 ± 337
**LNnT**	200 ± 290	65 ± 39	108 ± 76	285 ± 246	525 ± 315	446 ± 234	571 ± 321	250 ± 188
**LNFP I**	350 ± 450	180 ± 140		788 ± 754	1228 ± 557	945 ± 436	1036 ± 492	650 ± 416

**LNFP II**	2770 ± 2140			1853 ± 879				
**LNFP III**				92 ± 51				
**LNFP V**		25 ± 25						
**3′-SL**	133 ± 56	79 ± 20	198 ± 59	361 ± 231	126 ± 36	127 ± 39	135 ± 51	134 ± 69
**6′-SL**	189 ± 265	78 ± 40	120 ± 45	162 ±128	97 ± 33.2	83 ± 54	84 ± 34	91 ± 108
**LST a**	44 ± 62				36 ± 23	33 ± 17	84 ± 55	11 ± 8
**LST b**	193 ± 215			118 ± 69				
**LST c**	201 ± 316			43 ± 42	56 ± 41	47 ± 58	145 ± 160	
**DSLNT**	317 ± 409			315 ± 246				

**(C3) Mature milk**	**4 months**					**6 months**		
**Reference**<br>**Year**	[42]<br>2010	[32]<br>2016	[30]<br>2017	[27]<br>2018	[25]<br>2018	[25]<br>2018	[25]<br>2018	[23]<br>2019
**Company**		Nestle	Nestle		Fonterra	Fonterra	Fonterra	Fonterra
**Country**	Samoa (22-155d)	China (2-4 months)	Singapore	Canada (Caucasian and Asian mothers) (3-4 months)	China	China	Malaysia	UAE
**3′-GOS/3'-GL**								
**6′-GOS /6'-GL**								
**DFLac**				314 ± 261				
**A-tetra**		18 ± 15						
**FLNH/FLNH I/FLNH II**				90 ± 68				
**TFLNH**								
**DFLNT**				1440 ± 770				
**LNnFP**		8.2 ± 4.1						

**LNnFP V**						
**LNH**		72 ± 44.0				
**LNnH**	50 ± 20					
**LNnDFH**						
**MFLNH I/2’-FLNH**						
**MFLNH III/3-FLNH**						
**2'3-DF-LNH**						
**DFLNH/DFLNHa/DFLNH I/DFLNH II**		70 ± 87				
**DFLNnH**						
**DSL**			0.3 ± 0.6	0.0 ± 0.1		0.2 ± 0.4
**SLNFP I**						
**3'S3FL**			9.1 ± 7.5	10.5 ± 6.9	9.0 ± 6.5	10 ± 14
**SLNFP II**						
**6'SLN**			4.0 ± 2.3	2.0 ± 2.1	3.6 ± 2.8	5 ± 1
**DSLNH**		113 ± 91				
**FDSLNH**		655 ± 429				
